# *Aerococcus urinae* Endocarditis: Not So Severe After All?

**DOI:** 10.1177/2324709619852773

**Published:** 2019-06-06

**Authors:** Torgny Sunnerhagen, Magnus Rasmussen

**Affiliations:** 1 Lund University, Lund, Sweden

Dear Editor,

We read with interest the report by Adomavicius et al,^1^ describing a case of mitral valve infective endocarditis (IE) caused by *Aerococcus urinae.* There are 4 statements and conclusions in the article that make us concerned. The first statement is that IE caused by aerococci is a condition with a very severe course of disease. This conclusion by the authors is based on a collection of case reports listed in their report. When we studied all cases of *A urinae* IE reported to the Swedish Endocarditis Registry between 2002 and 2014, however, we found 14 cases of whom none had a fatal outcome.^2^ In a case series of aerococcal bacteremia (some of whom had IE), the overall mortality was 6%, with 2 out of 5 patients with IE leading to the death of the patient (1 died at home 90 days after blood culture positivity).^3^ The second conclusion concerns the risk of embolism in aerococcal IE, which in the report by Adomavicius et al is claimed to be high.^1^ While cerebral embolization is a serious complication of IE, we do not agree with the authors that this is a common complication of aerococcal endocarditis. When all reported Swedish cases were investigated, embolism was found in only 1 case in 14 of *A urinae* IE cases.^2^ The third statement that is problematic is when the authors recommend that surgical treatment should be performed as quickly as possible. In light of the fact that IE caused by aerococci does not seem to have a higher rate of death or more embolisms than IE caused by other bacteria, we find it appropriate to recommend surgery according to the same indications as for IE caused by other pathogens. In cases where surgery is deemed necessary, it should of course be done without delay. The fourth and last point concerns the recommendation put forward by Adomavicius and coworkers that a combination of an aminoglycoside and a β-lactam antibiotic should be used to treat aerococcal IE. The use of aminoglycoside combinations have limited clinical evidence. In vitro results show bactericidal synergy against aerococci in some isolates but failed to show that for other isolates.^2^ We thus find the recommendation to combine these antibiotics without firm support in the literature.

As pointed out also by Adomavicius et al,^1^ there is a risk that only dramatic cases of IE are published as case reports. Therefore, it is essential that results also from larger case series are reported and considered before drawing conclusions about aerococcal IE. From the current evidence, we conclude that the prognosis, and proposed treatment of aerococcal endocarditis, should be relatively similar to that of IE caused by, for example, α-hemolytic streptococci. IE is a severe type of infection, but there is no evidence that IE caused by aerococci has a particularly unfavorable prognosis.
